# Clinical and laboratory characteristics of patients hospitalised with COVID-19: clinical outcomes in Abu Dhabi, United Arab Emirates

**DOI:** 10.1186/s12879-022-07059-1

**Published:** 2022-02-08

**Authors:** Mariam Al Harbi, Nawal Al Kaabi, Asma Al Nuaimi, Jehad Abdalla, Tehmina Khan, Huda Gasmelseed, Asad Khan, Osama Hamdoun, Stefan Weber

**Affiliations:** 1 Corporate Academics and Research Affairs, Abu Dhabi Health Services (SEHA), Abu Dhabi, United Arab Emirates; 2 Infection Control Chair and Chief Medical Officer, Sheikh Khalifa Medical City, Abu Dhabi Health Services (SEHA), Abu Dhabi, United Arab Emirates; 3 Infectious Disease Department, Al Rahba Hospital, Abu Dhabi Health Services (SEHA), Abu Dhabi, United Arab Emirates; 4 Infectious Disease Department, Sheikh Khalifa Medical City, Abu Dhabi Health Services (SEHA), Abu Dhabi, United Arab Emirates; 5 Infectious Disease Department, Al Ain Hospital, Abu Dhabi Health Services (SEHA), Abu Dhabi, United Arab Emirates; 6 Infectious Disease Department, Tawam Hospital, Abu Dhabi Health Services (SEHA), Abu Dhabi, United Arab Emirates; 7 Department of Pediatrics, Al Ain hospital, Abu Dhabi Health Services (SEHA), Abu Dhabi, United Arab Emirates; 8 Department of Laboratory and Pathology, Sheikh Khalifa Medical City, Abu Dhabi Health Services (SEHA), Abu Dhabi, United Arab Emirates

**Keywords:** COVID-19, Clinical features, Disease severity, Mortality, Outcome

## Abstract

**Background:**

Severe acute respiratory syndrome coronavirus 2 (SARS‐CoV‐2) was first reported in December 2019. The severity of coronavirus disease 2019 (COVID-19) ranges from asymptomatic to severe and potentially fatal. We aimed to describe the clinical and laboratory features and outcomes of hospitalised patients with COVID-19 within the Abu Dhabi Healthcare Services Facilities (SEHA).

**Methods:**

Our retrospective analysis of patient data collected from electronic health records (EHRs) available from the SEHA health information system included all patients admitted from 1 March to 31 May 2020 with a laboratory-confirmed PCR diagnosis of SARS-CoV-2 infection. Data of clinical features, co-morbidities, laboratory markers, length of hospital stay, treatment received and mortality were analysed according to severe versus non-severe disease.

**Results:**

The study included 9390 patients. Patients were divided into severe and non-severe groups. Seven hundred twenty-one (7.68%) patients required intensive care, whereas the remaining patients (92.32%) had mild or moderate disease. The mean patient age of our cohort (41.8 years) was lower than the global average. Our population had male predominance, and it included various nationalities. The major co-morbidities were hypertension, diabetes mellitus and chronic kidney disease. Laboratory tests revealed significant differences in lactate dehydrogenase, ferritin, C-reactive protein, interleukin-6 and creatinine levels and the neutrophil count between the severe and non-severe groups. The most common anti-viral therapy was the combination of Hydroxychloroquine and Favipiravir. The overall in-hospital mortality rate was 1.63%, although the rate was 19.56% in the severe group. The mortality rate was higher in adults younger than 30 years than in those older than 60 years (2.3% vs. 0.95%).

**Conclusions:**

Our analysis suggested that Abu Dhabi had lower COVID-19 morbidity and mortalities rates were less than the reported rates then in China, Italy and the US. The affected population was relatively young, and it had an international representation. Globally, Abu Dhabi had one of the highest testing rates in relation to the population volume. We believe the early identification of patients and their younger age resulted in more favourable outcomes.

## Background

Coronavirus disease 2019 (COVID‐19), caused by severe acute respiratory syndrome coronavirus 2 (SARS‐CoV‐2), was first reported in December 2019 [1]. The severity of COVID-19 ranges from asymptomatic to severe infection leading to death [1]. On March 11, 2020, the World Health Organization (WHO) announced the emergence of a new SARS-CoV-2 virus pandemic [2]. Meanwhile, the United Arab Emirates (UAE) officially reported its first case of COVID-19 on January 29, 2020 [3]. By the end of the first wave of the pandemic in the UAE by 14 August 2020, the estimated total number of COVID-19 cases was 63,819 with 359 deaths, and 5,851,453 tests had been done, covering nearly 60% of the population [4, 5]. Globally, the total number of cases is estimated to exceed 18 million, including more than 800,000 deaths [2].

Abu Dhabi Health Services (SEHA) is the largest healthcare provider in UAE consisting of 13 hospitals and 46 clinics. It serves the western, eastern and middle regions of Abu Dhabi, encountering approximately 5 million patients per year through in-patient and out-patient services [5]. Initially 6 hospitals were designated for COVID-19 cases however with the high surge of cases, the remaining hospitals designated COVID-19 wards depending on the need. All SEHA facilities follow the National Guidelines for Clinical Management and Treatment of COVID-19 unified by all health regulatory bodies issued by the UAE Ministry of Health and Prevention [6, 7]. The UAE has the highest testing rate globally for COVID-19 relative to the population which facilitated the widespread screening for SARS-CoV-2 [5, 8].

Abu Dhabi is the capital of the UAE and reported to have a unique young demographic and a high percentage of expatriate residents compared to local citizens. In the 2019 statistical yearbook report from The Statistics Center of Abu Dhabi (SCAD), the population of Abu Dhabi as of mid-2016 was 2,908,173 and it reported the majority of people falling within the age range of 15–59 years [9]. In a 2016 analysis, Emirati citizens constituted 19% of the Abu Dhabi population, with the remaining citizens being expatriates [9]. The most common clinical symptoms of COVID-19 reported during the first wave of the disease were fever, cough, fatigue, headache and gastrointestinal symptoms such as vomiting and diarrhoea, whereas the most common laboratory findings were elevated C-reactive protein (CRP) and lactate dehydrogenase (LDH) levels and decreased lymphocyte counts. Additionally, Interleukin-6 (IL-6) a pro-inflammatory marker was anticipated to have a major role in predicting the progression and severity of COVID-19 disease [10]. Specific radiological features were noted on computed tomography, namely bilateral pneumonia and ground-glass opacity [11]. Children have less severe symptoms and much better outcomes than adults do. However, children may rarely present with multi-system inflammatory syndrome [12]. Significant differences in the clinical and demographic features of patients with COVID-19 have been noted in different regions of the world [13]. Most studies about COVID-19 were conducted in non-Arab countries [14], and few studies have highlighted the differences of clinical profiles, management and outcomes of patients with COVID-19 in the Middle East and Gulf region [15]. UAE National guidelines for the treatment of pneumonia and acute respiratory distress syndrome caused by SARS-CoV-2 were followed [6], and for intensive care management, the national guidelines for critical care were followed [7]. The outcomes of patients treated for COVID-19 in the UAE have not been reported in the literature. Anecdotally, patients in the UAE are faring better than their Western counterparts, but no study has analysed the available data to validate this supposition.

In this study, we analysed the clinical features, laboratory markers and patient outcomes of hospitalized patients with COVID-19 during the first wave of the pandemic in Abu Dhabi, UAE.

## Methods

### Study design

We used retrospective patient data collected from electronic health records (EHRs) available from the SEHA health information system which included all adult (≥ 18 years old) patients admitted from 1 March to 31 May 2020 with a Polymerase Chain Reaction (PCR) confirmed diagnosis of SARS-CoV-2 infection on nasal swabs. We excluded paediatric patients and any patient admitted with an ongoing COVID-19 diagnosis outside the study period.

### Study groups

The patients were assigned to the severe or non-severe group based on the level of care received. Patients who required intensive care unit (ICU) or high dependency unit (HDU) admission or mechanical ventilation were assigned to the severe group, and all other patients comprised the non-severe group.

### Data collection and variables

We collected data on hospitalisations and intensive care admissions from Abu Dhabi Health Services (SEHA). We gathered electronic data from six major hospitals.

The data extraction process was based on patient documentation in EHRs and handled by the Cerner team located in the UAE in addition to SEHA Health Information System application analysts. The following data were collected in this study: (1) demographic data including age, gender and nationality; (2) past medical history including co-morbidities based on ICD-10 codes in the patients’ EHRs (diabetes mellitus [DM], hypertension [HTN], renal disease, heart disease, asthma, chronic obstructive pulmonary disease [COPD], malignancy and pregnancy), as well as lifestyle variables such as smoking, exercise habits and diet; (3) assessments recorded at admission (vital signs, Sequential Organ Failure Assessment [SOFA] score, Modified Early Warning Score [MEWS] and Glasgow Coma Score [GCS]) to reflect clinical severity in addition to symptoms such as fever, cough, sore throat, headache and vomiting and diarrhoea and (4) all laboratory values available in the EHRs collected within the first 24 h after admission. (5) Outcome measures included the status of the patient (recovered or died), duration of admission in days (length of stay) and viral clearance defined as the time (measured in days) from hospital admission to the first negative SARS-CoV-2 PCR laboratory result. (6) The types of medications used by all patients were also generally described without any correlation to the severity of COVID-19. Because of the large amount of data included in the study and the nature of descriptive studies, we did not perform any imputation for missing data. Data were analysed with no adjustment for any missing values or variables.

### SARS-CoV-2 detection

SARS-CoV-2 was detected by real-time reverse transcription-PCR via detection of the N and ORF1ab genes using a U-Top COVID-19 Detection Kit (Seasun Biomaterials, Daejeon, Korea) or the E and S genes using a RealStar PCR kit (Altona Diagnostics, Hamburg, Germany). The results were interpreted according to the manufacturer’s recommendation.

### Statistical analysis

Baseline characteristics were summarised using descriptive statistics, including the mean, median, interquartile range (IQR) and standard deviation (SD) for continuous measures and frequency tables for categorical variables. Categorical variables were compared using the chi-squared test or Fisher’s exact test, and continuous variables were compared using the unpaired *t*-test or its non-parametric equivalent. Statistical significance was indicated by P < 0.05 (two-sided). The data analysis was performed using STATA statistical software version 12.0.

The data were further analysed using time to event analysis (survival analysis). Kaplan–Meier estimates were used to compare survival between the groups based on the top three co-morbidities observed in the cohort, namely DM, HTN and renal disease. The survival time was the duration from the date of admission to that of death. In this study, patients lost to follow-up were considered as right censored. Discharge from the hospital will be considered as a competing event. Survival curves of different comorbidities were compared using the equivalent of log-rank test in the case of competing events.

### Ethic approval

Ethics approval and consent to participate approval were obtained from the National COVID-19 Institutional Review Board committee on June 6, 2020 (reference number: CVDC-10-06/2020-10-1). The requirement for informed consent was waived for this research because of the nature of the study, which is a retrospective chart review of unidentified data.

## Results

### Demographics

A total of 9390 patients were admitted to SEHA hospitals in Abu Dhabi during our study period, of which 721 (7.68%) required either ICU (455 patients) or HDU (266) admissions (severe group), while the remaining 8669 patients (92.32%) with mild-to moderate symptoms were admitted to either regular wards at designated SEHA hospitals. Patients allocated to isolation hotels were not included in this study as the purpose of keeping them was quarantine. The baseline demographics of the study population is detailed in Table 1. The mean patient age was 41.8 ± 11.89 years (95% CI 41.61–42.09). In total, 18.3% were aged 19 to 29 years, 74.6% were aged 30 to 60 years and the remaining 7.1% were older than 60 years. Patients with severe disease were significantly older than those with non-severe disease (p < 0.001). The male-to-female ratio was 4.9:1.

**Table 1:** Demographics of patients hospitalised with COVID-19

	Overall	Severe	Non-severe
Demographics	N = 9390	N = 721 (7.68%)	N = 8669 (92.32%)
Mean age, years (mean ± SD)	41.85 ± 11.89	44.75 ± 13.34	41.61 ± 11.75
Sex, N (%)			
Female	1598 (17.02%)	133 (18.45%)	1465 (16.9%)
Male	7792 (82.98%)	588 (81.55%)	7204 (83.1%)
Nationality, N (%)			
Emirati	688 (7.33%)	89 (12.34%)	599 (6.9%)
Non-Emirati	8702 (92.67%)	632 (87.66%)	8070 (93.1%)
Indian	3673 (39.12%)	232 (32.18%)	3441 (39.7%)
Bangladeshi	945 (10.06%)	69 (9.57%)	876 (10.1%)
Pakistani	1274 (13.57%)	102 (14.15%)	1172 (13.5%)
Pilipino	760 (8.09%)	52 (7.21%)	708 (8.2%)
Egyptian	371 (3.95%)	24 (3.33%)	347 (4%)
Nepalese	358 (3.81%)	18 (2.50%)	340 (3.9%)
Others	1321 (14.068%)	135 (18.7%)	1186 (13.7%)

The most common nationality was Indian (39.12%), followed by Pakistani (13.57%), Bangladeshi (10%), Filipino (8%) and Emirati (7.3%). The remaining 14% of patients were from 21 different nationalities across the globe.

### Admission assessment and co-morbidities

All patients were assessed within the first 24 h of hospitalisation. The most common symptoms reported by patients are listed in Table 2. The range of vital signs values reflected the spectrum of severe COVID-19 even though most patients had normal vital signs. Further, 106 patients required mechanical ventilation within the first 24 h of hospitalisation. The SOFA score, MEWS and GCS in patients admitted with COVID-19 is presented in Table 3, all three scores differed between the groups (all P < 0.0001).

**Table 2:** Clinical Features and respiratory support for hospitalized COVID-19 patients

Signs and symptoms	N (%)
Cough	3156 (51.86%)
Vomiting or diarrhoea	177 (1.88%)
Sore throat	871 (24.83%)
Shortness of breath	715 (7.61%)

Vital signs	Median (IQR)
Temperature (centigrade)	36.8 (36.7–37.1)
Respiratory rate (bpm)	18 (18–20)
Heart Rate (bpm)	83 (75–92)
Systolic blood pressure (mmHg)	132 (122–143)
Diastolic blood pressure (mmHg)	83 (75–90)
Oxygen saturation (%)	99 (97–99)
Oxygen flow rate (L/min)	2 (0–3)

Respiratory support	N (%)
Mechanical ventilation	106 (1.42%)
Oxygen therapy (any form)	386 (5.16%)

**Table 3:** Clinical severity scores and co-morbidity rates in hospitalised patients with severe and non-severe COVID-19

	Overall N = 9390	Severe N = 721	Non-severe N = 8669	P-value
MEWS Median (IQR)	0 (0–0)	4 (2–6)	0 (0–0)	< 0.0001
SOFA score Median (IQR)	0 (0–0)	0 (0–3)	0 (0–0)	< 0.0001
Glasgow Coma Scale Median (IQR)	14.9 (3–15)	15 (15–15)	15 (15–15)	< 0.0001
Co-morbidities^a^	2706 (28.8%)	542 (75.17%)	2164 (24.93%)	
Diabetes mellitus	962 (10.24%)	195 (27.05%)	767 (8.85%)	< 0.0001
Hypertension	1028 (10.94%)	237 (32.87%)	791 (9.12%)	< 0.0001
COPD	3 (0.03%)	1 (0.14%)	2 (0.02%)	0.095
Asthma	176 (1.87%)	16 (2.22%)	160 (1.85%)	0.477
CAD	89 (0.95%)	16 (2.22%)	73 (0.84%)	0.0002
Ischaemic heart disease	93 (0.99%)	9 (1.25%)	84 (0.97%)	0.466
CKD	166 (1.76%)	41 (5.69%)	125 (1.44%)	< 0.0001
Malignancy	189 (2.01%)	27 (3.74%)	162 (2.40%)	0.0005
Pregnancy	40 (0.43%)	4 (0.5%)	36 (0.4%)	0.580
Body mass index Median (IQR)	26.22 (23.83–29.34)	25.99 (23.88–29.48)	26.23 (23.83–29.29)	0.589

^a^Presence of one or more co-morbidity

The comparison of co-morbidities with patients from the severe and non-severe groups is listed in Table 3. A total of 75% in the severe group had at least one co-morbidity from the three major co-morbidities ie HTN (32.8%), DM (27%) and chronic kidney disease (CKD 8 5.6%). Only 25% had a co-morbidity among patients with non-severe COVID-19 with HTN being the predominant condition. The rates of HTN, DM and CKD, in addition to coronary artery disease (CAD) and malignancy, differed significantly between the groups (DM, HTN, CKD p < 0.0001, CAD p = 0.0002 and malignancy p = 0.0005). The medical records lacked documentation regarding physical activity for most admitted patients, and smoking habits were not accurately reflected. No inference can be made based on existing data listed in the medical records to determine the effect diet and physical exercise parameters between the groups. The mean body mass index (BMI) was not significantly higher in the severe group (p = 0.5895). Chronic obstructive pulmonary disease (COPD) and asthma had a low frequency compared to other co-morbid conditions.

### Laboratory values

We found statistically significant differences in lymphocyte (p = 0.005) and neutrophil counts (p < 0.0001) but not in the total white blood cell count (Table 4). The levels of certain inflammatory markers, such as ferritin, CRP and LDH, were also significantly different between the severe and non-severe groups with each having p < 0.0001). Additionally, the levels of IL-6, significantly differed between the groups (p < 0.0001).

**Table 4:** Laboratory Markers in hospitalised COVID-19 patients with the severe and non-severe groups

Variables	Overall (mean ± SD)	Severe (mean ± SD)	Non-severe (mean ± SD)	P-value
While blood cell count (× 10^9^/L)	6.76 ± 3.69	7.02 ± 3.98	6.74 ± 3.66	0.0635
Lymphocyte count (× 10^9^/L)	1.86 ± 2.72	1.79 ± 0.80	1.89 ± 2.84	0.0056
Neutrophil count (× 10^9^/L)	4.22 ± 2.36	4.73 ± 03.71	4.17 ± 2.17	< 0.0001
Creatinine (µmol/L)	88.32 ± 85.61	104.78 ± 124.10	86.72 ± 80.73	< 0.0001
HbA1c (%)	7.81 ± 2.50	8.42 ± 2.38	7.77 ± 2.50	0.0591
LDH (IU/L)	264.46 ± 128.50	310.10 ± 182.50	259.76 ± 120.62	< 0.0001
Ferritin (mcg/L)	555.95 ± 821.86	768.05 ± 1003.73	534.46 ± 798.09	< 0.0001
CRP (mg/L)	29.51 ± 53.16	56.55 ± 86.87	26.83 ± 47.75	< 0.0000
IL-6 (pg/mL)	158.84 ± 734.08	472.65 ± 1508.39	78.46 ± 255.48	< 0.0000

### Outcomes

Among 9390 patients admitted to our healthcare network, 158 patients died from direct causes related to COVID-19. The overall in-hospital mortality rate was 1.68%. The mortality rate was 19.56% in the severe group, compared to 0.2% in the non-severe group (p < 0.0001). The mortality rate in patients younger than 30 years was 2.33%, while it was 1.6% in patients aged 30 to 60 years. However, only six patients older than 60 years died (0.9%) (summarized in Table 5).

**Table 5:** Outcomes among hospitalized patients with COVID-19

	Overall N = 9390	Severe N = 721	Non-severe N = 8669	P-Value
Mortality rate N (%)	158 (1.68%)	141 (19.56%)	17 (0.2%)	< 0.0001
Length of stay (days, mean ± SD)	6.46 ± 5.27	7.42 ± 6.65	6.36 ± 5.12	< 0.0001
Viral clearance^a^ (days, mean ± SD)	13.22 ± 10.19	15.41 (± 10.31)	13.05 ± 10.17	< 0.0001

^**a**^Viral clearance was defined as the number of days from admission to the first negative SARS-CoV-2 PCR result

The mean length of hospital admission for all patients was 6.4 ± 5.27 days, with the longest stay being 65 days. The mean duration was 7.42 ± 6.65 days in the severe group compared to that in the non-severe group of 6.36 ± 5.12 days (p < 0.0001). The mean ICU admission was 7.42 ± 6.65 days. The mean viral clearance was 13.22 ± 10.19 days, with the upper limit being 68 days, while 95 patients (50%) remained PCR positive until death. The mean viral clearance in the non-severe group was 13.05 days while it was 15.41 days in the severe group (p < 0.0001).

**Fig. 1:**
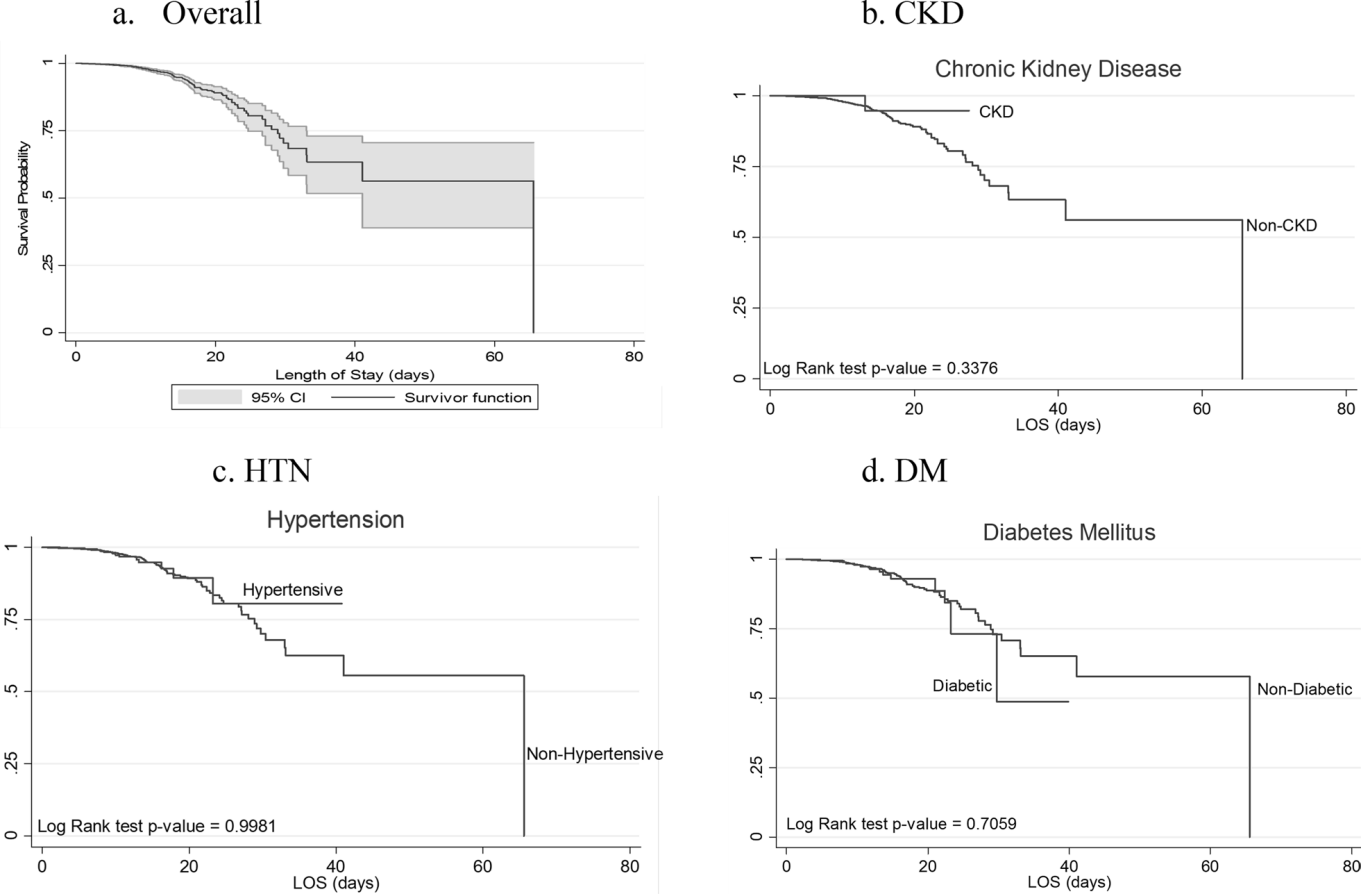
Kaplan–Meier Survival analysis on patients admitted to ICU. **a** Without comorbidities and with (**b**) DM, **c** HTN and **d** CKD

Survival analysis conducted on patients admitted to ICU with co-morbidities such as DM, HTN and CKD showed no significant differences compared to patients without these conditions (Fig. 1).

### Treatment

The drugs used as antiviral treatment are listed in Table 6. More than 50% of patients received two or more drugs. The most commonly used treatment was the combination of Favipiravir and Hydroxychloroquine (HCQ) with 63% receiving HCQ. The next commonest drug used was Camostat mesylate (11.65%) and the least frequently used was Lopinavir/Ritonavir (9.04%). Tocilizumab was only used in patients with elevated IL-6. Only 2% were given steroids (dexamethasone, methylprednisolone sodium succinate and prednisolone). The use of anticoagulants was initiated in the middle of March 2020 with 47.4% receiving low-molecular-weight heparin (Enoxaparin Sodium) [16]. Some hospitals used Doxycycline as a presumptive treatment for COVID-19. Per the clinical protocol and UAE guidelines, medical treatment was offered to all symptomatic patients.

**Table 6:** Medications used in hospitalised patients with COVID-19

Medications	N (%)
HCQ	5945 (63.31%)
Favipiravir	5057 (53.86%)
Enoxaparin	4359 (46.42)
Camostat	1094 (11.65%)
Lopinavir/ritonavir	849 (9.04%)
Azithromycin	595 (6.34%
Chloroquine	260 (2.77%)
Tocilizumab	23 (2.5%)
Steroids^a^	188 (2%)
Doxycycline	104 (1.1%)
Heparin	102 (1%)

^a^Steroids: dexamethasone, methylprednisolone Na succinate and prednisolone

## Discussion

The mean age of our cohort was 41.8 ± 11.89 years and the group consisted of 7.33% Emiratis and 92.67% non-Emiratis. Males were 82.98% and 17.02% were females. Only 7.68% of our patients required ICU admission. 28.8% of patients had one or more comorbidity and the three common ones were DM, HTN and CKD. Laboratory tests revealed significant differences in LDH, ferritin, CRP, IL-6, creatinine levels and the neutrophil count between the severe and non-severe groups. The most common anti-viral therapy was the combination of Hydroxychloroquine and Favipiravir. The overall in-hospital mortality rate was 1.63%, although the rate was 19.56% in the severe group. The mortality rate was higher in adults younger than 30 years than in those older than 60 years (2.3% vs. 0.95%).

Comparing the mean age of our cohort (41.8 years) to others, it was lower than the mean age (65.5 years) reported in the United States [16] while China has reported variable mean ages in different studies ranging from 45 to 59 years [11]. This study reflected the actual population of the UAE which is a heterogeneous mixture of different nationalities in comparison to data published from China and Western countries [9, 13] mostly reflecting non-Emiratis (92.67%) of whom Indians (39.12%) and Pakistanis (13.57%) were the largest two nationalities. The male predominance of cases followed global trends [13, 16] and reflected the actual community of Abu Dhabi [9]. The ICU admission rate in Abu Dhabi was considered lower than that global rates where 20–30% of COVID-19 admissions required intensive care [17]. The three common comorbidities namely DM, HTN and CKD were significantly different among the severe and non-severe groups (p < 0.0001). This was similar to the common comorbidities observed in US, Italy and China [13, 18]. We detected significant differences in laboratory markers such as Ferritin, CRP, LDH, LI-6, creatinine and lymphocyte count in the severe compared to the non-severe group, suggesting a role identifying clinical severity. These markers are reported in many counties like US, China and Italy to play a role in severe COVID-19 [13, 16, 19]. Elevated creatinine levels specifically suggest pre-existing renal disease or the risk of developing acute renal failure secondary to COVID-19 [20]. There is emerging evidence that the neutrophil–lymphocyte ratio is a predictor of severity in patients with COVID-19 [21]. These laboratory assessments might represent predictive modalities for severity of COVID-19 [19].

The overall mortality (1.68%) was favourable in our patients with a low rate (0.9%) for patients older than 60 years. The mortality rate (19.56%) in the severe group was relatively lower than in other studies [22, 23]. However, the mortality rate was unexpectedly higher in our younger patients. Some studies looked into risks predisposing young adults to severe COVID-19 and death of which smoking, obesity and the presence of at least one comorbidity were reported as risk factors [24, 25].

Our analysis represents data from hospitalised patients in Abu Dhabi. However, the indication for hospitalisation changed during the study period. Initially, all people with a positive PCR were admitted regardless of clinical presentation for isolation and monitoring. Subsequently, as incidence increased and public awareness of self-isolation improved, only the ill patients were hospitalized; asymptomatic patients and Persons Under Investigation (PUI) were isolated in hotels [6, 26]. Many countries were revising the management protocols of COVID-19 and worked on optimising the resources to manage the surge of COVID-19 cases especially the indications of ICU admission [27, 28].

We believe the results of this study were likely affected by the fact a certain percentage of patients in the non-severe group had asymptomatic or mild disease. The data represented a younger population, which is characteristic of UAE’s demographics.

Since the beginning of the pandemic, medical care has been offered to the entire UAE population for free including all patients regardless of their insurance coverage [6, 26, 27]. Similarly, many countries were implementing local policies to assure health equity especially to minority groups [29]. Despite this policy, the data indicate that 21 patients who required mechanical ventilation (106 patients) during the first 24 of admission died within one week. Some nationalities like Indians (39.12%) and Pakistani (13.57) representing the majority of blue-collar workers might not have been aware that treatment was free which may have resulted in late hospital presentation [30]. Although symptom duration prior to admission cannot be verified due to the EHRs lacking detail, the need for mechanical ventilation on admission in 106 (1.42%) patients does support our assumption [31].

The country’s infectious disease experts have developed clinical and intensive care guidelines based on the best available evidence [6, 7]. These guidelines were updated six times during the preparation of this manuscript. At the beginning of the pandemic, treatment primarily focused on the use of HCQ and Lopinavir/ritonavir. Subsequently, with the anecdotal observation of ineffectiveness and evolving evidence from China and Japan, Favipiravir was added [32]. Although the combination of HCQ and Favipiravir was the most common treatment in this study, we cannot conclude whether these treatments improved the likelihood of recovery in this population. The treatment options in UAE’s COVID-19 national guidelines was similar to WHO treatment guidelines for COVID-19 and updates in treatment options such as the use HCQ was changed with the available evidence and international guidelines [33].

Anti-coagulation was another commonly used treatment strategy [34], and the therapy was adjusted according to the coagulation profile of the patients using prophylaxis guidelines [6, 33]. This practice was started in mid-March 2020 across SEHA hospitals (based on the critical care counsel at SEHA recommendation after evidence suggested the existence of a hypercoagulable state in COVID-19) [34, 35].

Our study has several limitations. Firstly this is a retrospective descriptive study using existing EHRs’ data which may not be an optimal data source. Data with inadequate or variable documentation based on users in the EHRs had to be excluded from our analysis. This is a common limitation reported in retrospective data in other countries as well [13, 16]. Secondly, the study period included patients admitted during the first wave of the pandemic which was a period of frequent changes in indications for hospitalization. Thirdly, the study period included patients admitted during the first wave of the pandemic which was a period of continuous change of management plans and protocols. However, there are several strengths that gives value to this study. It is one of the first observational studies to characterise COVID-19 patients in the UAE in addition to reporting the favourable disease outcomes in Abu Dhabi that highlights the successful strategy used in the country to battle the pandemic especially with the widespread PRC testing rate and free healthcare access to all residents regardless of their insurance coverage or nationality. Additionally, it identifies key features observed in severe COVID-19 disease like common co-morbidities and laboratory markers.

Many studies have investigated the role of different pharmacological agents permitted for emergency use during the initials phase of the pandemic which contributed to establishment of the recent COVID-19 management guidelines [6, 32, 36–38]. Key features like comorbidities and certain laboratory values have been identified and included in the recent guidelines for management of COVID-19 [17, 19, 33].

## Conclusions

We have shown that patients with COVID-19 in the UAE in Abu Dhabi had relatively low morbidity and mortality rates and high recovery rates. The presence of DM, HTN and CKD did not affect patients’ outcome in the ICU. The UAE has the highest testing rate globally for COVID-19 relative to the population which facilitated the widespread screening for SARS-CoV-2 possibly leading to the early identification of cases and relatively better outcomes. Both the early identification of cases and the younger age of the population contributed to the relatively favourable outcomes. However, the lower mean age at the time of death highlights the need for increased awareness among younger people.

## Data Availability

The data that support the findings of this study are available from the Health Information System department at Abu Dhabi Health Services, but restrictions apply to the availability of these data, which were used under licence for the current study, and so are not publicly available. Data are, however, available from the authors upon reasonable request and with permission of Abu Dhabi Health Services and the Abu Dhabi Department of Health.

## Abbreviations

SARS‐CoV‐2: Severe acute respiratory syndrome coronavirus 2
COVID-19: Coronavirus disease 2019
UAE: United Arab Emirates
EHR: Electronic Health Records
PCR: Polymerase chain reaction
CAD: Coronary artery disease
CKD: Chronic kidney disease
COPD: Chronic obstructive pulmonary disease
CRP: C-reactive protein
DM: Diabetes mellitus
GCS: Glasgow Coma Score
HDU: High dependency unit
ICU: Intensive care unit
IQR: Interquartile range
IRB: Institutional Review Board
MEWS: Modified Early Warning Score
PCR: Polymerase chain reaction
SCAD: Statistics Center of Abu Dhabi
SD: Standard deviation
SOFA: Sequential Organ Failure Assessment
UAE: United Arab Emirates
WHO: World Health Organization
BMI: Body Mass Index
